# Effect of Stirring on Organic Matter Conversion in Horizontal Biodigesters

**DOI:** 10.1002/wer.70343

**Published:** 2026-04-07

**Authors:** Gabrielle Oliveira Rosa da Cruz, André Pereira Rosa, Izabelle de Paula Sousa, Cláudio Leite de Souza, Alisson Carraro Borges

**Affiliations:** ^1^ Department of Agricultural Engineering Federal University of Viçosa Viçosa Brazil; ^2^ Department of Sanitary and Environmental Engineering Federal University of Minas Gerais Belo Horizonte Brazil

**Keywords:** anaerobic digestion, horizontal flow biodigester, mechanical stirring, organic matter conversion, swine wastewater

## Abstract

This study investigated the effect of localized mechanical stirring on the performance and organic matter conversion pathways in two bench‐scale horizontal flow anaerobic biodigesters treating swine wastewater (SW). Two reactors (total volume of 10.6L; working volume of 7.95 L) were operated in parallel for 150 days under identical conditions (hydraulic retention time [HRT] = 25 days; volumetric organic loading rate [VOLR] = 0.3–0.5 g V L^−1^ day^−1^ of TVS): one equipped with mechanical mixing (HF_w_) and the other operated without mixing (HF_w/o_). After system stabilization, no statistically significant differences were observed between the reactors with respect to hydrolysis and methanogenesis (%) or biogas production and composition. Mass balance analysis demonstrated that HF_w_ accumulated fewer solids within the reactor (5.5% of the applied chemical oxygen demand [COD]) compared with HF_w/o_ (13.6%). These findings indicate that mechanical stirring effectively mitigates sludge accumulation without compromising overall treatment performance or biogas generation.

## Summary

- Mixing did not affect hydrolysis, methanogenesis percentages, or overall biogas production.
- Biodigesters with stirring showed more efficient solid removal from the applied COD load.
- Localized mixing improves reactor maintenance without compromising treatment performance.

## Graphical abstract

The mechanical stirring effectively mitigates sludge accumulation without compromising overall treatment performance or biogas generation, promoting a reduced buildup of solids within the reactor.

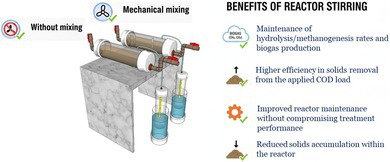

## 1 Introduction

Global pig consumption is projected to increase to 129 million tonnes over the next 10 years and account for one‐third of the total increase in meat consumption (OECD/FAO 2022). In 2025, Brazil remained the fourth largest producer of pork in the world, behind China, the European Union, and the United States. The country is expected to increase its production by 1% in 2026, accounting for 4.81 million tons of carcass. This growth is attributed to greater feed availability, stable domestic consumption, sustained external demand, and ongoing efforts to diversify export markets (USDA 2025).

However, the intensive pig farming industry has a high pollution potential, capable of compromising regional environmental resources. Among the impacts, the following stand out: contamination of surface and groundwater due to high concentrations of nutrients such as phosphorus and nitrogen (López‐Pacheco et al. 2021) and heavy metals, especially copper and zinc (Liu et al. 2020); the presence of hormones and antibiotics in digestate and sludge, which are used as fertilizers (Peng et al. 2017; Cheng et al. 2020; Lima et al. 2020; Gomes et al. 2022); as well as greenhouse gas (GHG) emissions and foul odors (Varma et al. 2021).

Anaerobic digestion is a widely used process for the treatment of agro‐industrial waste, such as wastewater from pig and cattle farming, slaughterhouse effluents, among others (Zubair et al. 2020). Its greatest advantage lies in the conversion of complex compounds into two main products: biogas (CH_4_ and CO_2_), which can be used as a source of energy and heat, and digestate, recognized as an efficient natural fertilizer due to the presence of nutrients such as nitrogen and phosphorus (Varma et al. 2021; Cândido et al. 2022; Hollas et al. 2022).

In tropical countries such as Brazil, covered lagoon biodigesters (CLB) have been widely employed to treat swine wastewater (SW) due to their low cost, ease of operation, high efficiency in odor reduction, and the potential for biogas‐based energy generation (Tápparo et al. 2021). However, the accumulation of solids remains one of the main challenges faced by this horizontal system (Sousa et al. 2022).

In many cases, physical (radial) mixing is an effective strategy to prevent the presence of dead zones and the local accumulation of inhibitory byproducts, as well as to enhance heat distribution and the contact between nutrients and the microbial community (Ghanimeh et al. 2020; Khoshnevisan et al. 2021). It also offers reduced maintenance requirements, lower contamination risks, and overall design simplicity (Shoshaa et al. 2024). Neuner et al. (2024) report a trend toward the use of mechanical mixing in biodigesters, related to better process control, lower operational costs, and easier maintenance compared to gas injection and, especially, impeller‐driven draft tube mixing.

The chemical oxygen demand (COD) mass balance can provide insights into the preferential pathways of organic matter degradation in anaerobic digestion—such as those observed in horizontal digesters—by considering the system's inputs and outputs (products) (Chernicharo 2019; de Souza 2010). In this context, Mazareli et al. (2016) reported that organic matter conversion in horizontal reactors depends primarily on biomass retention and the hydrodynamic regime, particularly when internal mixing is applied, as it alters solids distribution and mass transfer within the reactor. In this context, there is still a lack of integrated assessments of bench‐scale horizontal digesters treating SW that evaluate the influence of internal mixing and its impacts on operational performance and organic matter conversion pathways. This gap constrains the development of robust design and operational guidelines for such systems.

Therefore, this study aimed to evaluate the performance of anaerobic digestion and methane production in horizontal flow (HF) biodigesters treating SW, with the introduction of intermittent mechanical stirring. The specific objectives were as follows: (i) to assess the stability of the anaerobic digestion process in an automated biodigester using stability indicators (pH, IA/PA ratio), (ii) to evaluate operational aspects related to the adaptation of automated biodigesters, and (iii) to analyze the routes of organic matter conversion in automated biodigesters through COD mass balance.

## 2 Material and Methods

### 2.1 Experimental Setup

**FIGURE 1:**
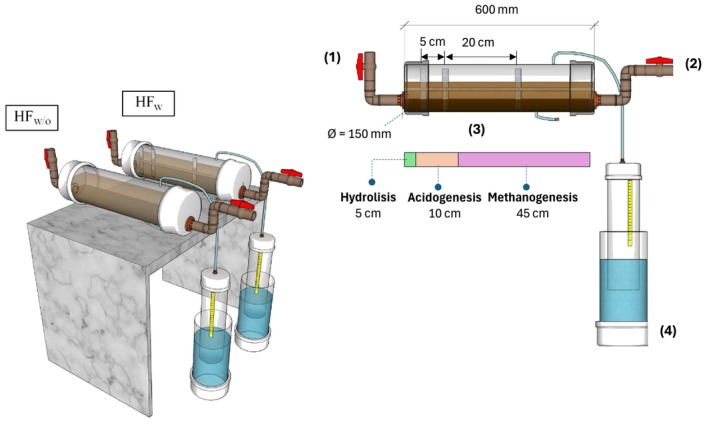
Detailed schematic of the horizontal flow biodigesters (1) inlet; (2) outlet; (3) impeller blades; (4) gasometer.

The experimental apparatus consisted of two bench‐scale horizontal flow biodigesters made of 0.60 m of 150 mm (ø) of PVC pipe, with total and working volumes of 10.60 and 7.95 L, respectively. The reactors were named HF_w_ and HF_w/o_, in which the first had a stirring mechanism to enhance biogas production and organic matter removal, while the second operated as a control, without mixing (Figure 1). The stirring set consisted of a 10‐mm (ø) shaft, coupled to three impeller blades (3) with dimensions of 60 mm × 30 mm, adapted from a study conducted by Khalil et al. (2021).

A direct current motor was connected to the unit to rotate the longitudinal shaft, and a valve was placed at the opposite end to withdraw the digestate (2). The system is fed through a valve (1). The biogas is collected from the top of the digester and sent to a gasometer for volume measurement.

Assuming plug‐flow behavior, with constant cross‐sectional area and uniform horizontal flow velocity, the hydraulic retention time increases linearly along the reactor length. Under these conditions, proportionality between process time and the distance traveled by the effluent can be assumed. Thus, a 60‐cm long digester was conceptually divided into the following zones: 5.0 cm for hydrolysis, 10 cm for acidogenesis, and 45 cm for methanogenesis. The local mixing was achieved using three rectangular paddles along the longitudinal axis, placed at 1.5, 5.0, and 20 cm away from the feeding end—those locations are assumed to coincide with the digestion stages. The shaft was continuously rotated at 25 rpm (Lindmark et al. 2014), activated four times per day, with a 6‐h interval and a duration of 2 min.

Each reactor was coupled to gasometers (4) made of PVC (internal and external diameters of 100 and 150 mm, respectively) to verify the daily production of biogas and methane. The quantification was based on the liquid displacement method, using saturated NaCl solution (Walker et al. 2009), with the aid of a graduated ruler (Figure 1).

Finally, the bench‐scale study was designed to preserve key operational features commonly found in full‐scale CLBs (e.g., HRT of 25 days, organic loading rates representative of SW, semicontinuous operation, and horizontal configuration). The objective was not to replicate full‐scale hydrodynamics but to provide qualitative trends and mechanistic insights into the influence of mixing on solids retention and organic matter conversion. Similar bench‐scale studies with reactor volumes below 10 L reporting trends relevant to full‐scale systems are available in the literature (Tampio et al. 2016).

### 2.2 Inoculation, Start‐Up, and Operational Conditions

The reactors were inoculated with anaerobic sludge from a full‐scale CLB, which has been operating continuously for nearly 5 years and treats the effluent from a swine production facility. The reactor working volume was initially filled with inoculum, followed by daily feeding with SW to allow biomass acclimation to the applied organic loading rate and to achieve steady‐state conditions. The inoculum was characterized by concentrations of 34,350 ± 5035 mg L^−1^ of TVS and 17,260 ± 421 mg L^−1^ of COD.

In this study, an acclimatization period of 50 days (corresponding to approximately 2 HRTs) was adopted, based on previous studies indicating that operation over roughly two hydraulic retention times (HRTs) can promote microbial acclimation and stable reactor performance (Arikan et al. 2015; Duan et al. 2019). Initially, the working volume of both reactors was filled with the inoculum, and daily, the SW was introduced to allow the biomass to adapt to the applied load and for the reactors to reach a steady state. During the acclimatization period, the reactors were not stirred to ensure that they started under the same conditions.

For the preparation of the SW, approximately 1 kg of solid swine manure was collected weekly from the maternity swine farming and frozen at −20°C until use. The reactors were fed every morning in a semicontinuous mode, with volumetric organic loading rate (VOLR) of 0.3–0.5g L^−1^ day^−1^ of TVS and HRT of 25 days, typical of CLBs (Kunz et al. 2019). The influent flow rate in each bench‐scale biodigester was 0.315 L day^−1^.

After acclimatization, the effect of stirring was evaluated over 100 operational days, divided into two phases: Phase I (Days 0–50), corresponding to the HF_w_ adaptation period and Phase II (Days 51–100), during which both systems displayed stable biogas production, corresponding to approximately two HRTs. The SW characteristics are shown in Table 1.

**TABLE 1:** Characteristics of SW used in the reactors.

Parameters	Mean ± SD (*n*)
pH	7.43 ± 0.48 (100)
VOLR as TVS (g L^−1^ day^−1^)	0.54 ± 0.21 (23)
Total alkalinity (mg L^−1^ as CaCO_3_)	910.55 ± 426.69 (20)
Total COD (mg L^−1^)	13,981 ± 3606 (20)
Soluble COD (mg L^−1^)	1698 ± 676 (22)
COD_s_/COD_t_	13.3 ± 6.6 (20)
TS (mg L^−1^)	17,116 ± 5999 (23)
TVS (mg L^−1^)	13,603 ± 5238 (23)
TVS/TS (%)	78.8 ± 6.6 (23)
TKN (mg L^−1^)	433.17 ± 18.09 (13)
NH_4_^+^‐N (mg L^−1^)	44.28 ± 5.42 (14)
TP (mg L^−1^)	468.53 ± 14.28 (13)

### 2.3 Analytical Methods

The pH and temperature of the influent and effluent SW were measured daily at the time of feeding, using a Hach HQ40D meter. The influent SW was analyzed in terms of total and soluble COD (COD_t_ and COD_s_), total and volatile solids (TS and TVS) (APHA 2023), and total alkalinity (TA) (Ripley et al. 1986). The sampling frequency was weekly until Day 39 and then twice a week.

The effluent SW was evaluated in terms of COD_t_ and COD_s_, TS and TVS (APHA 2023), and total (TA), partial (PA), and intermediate (IA) alkalinity (Ripley et al. 1986). The sampling frequency was three times a week (Days 0–29) and then twice a week (Days 30–100). Effluent SW samples were collected about 2 h before starting the mechanical stirring.

The inoculated sludge was characterized before being introduced into the biodigesters, in terms of COD, TS, TVS, and pH. At the end of the operation, the final sludge was also characterized for the same parameters in order to verify the biomass production during the evaluated period.

The biogas composition was analyzed weekly for methane content in a gas chromatograph equipped with a flame ionization detector (FID) (GC‐2014, Shimadzu Corporation). Each biogas sample (about 100 μL) was injected into the equipment at a flow rate of 25 mL min^−1^, using helium as carrier gas. The temperature of the injector was set at 120°C, while that of the FID was set at 200°C.

The volume of biogas produced in the reactors was quantified daily based on the displacement of PVC gas holders, multiplied by the cross‐sectional area (0.00785 m^2^). The measured volume was corrected to standard temperature and pressure conditions as established by the IUPAC (273.15 K and 100 kPa). Ambient temperature was monitored using a mercury column thermometer, with values ranging from 19°C to 24°C. The vapor pressure of the solution was determined using the Goff–Gratch, and the corrected biogas volume was calculated according to Equation (1) proposed by Parajuli (2011).

(1) $$V_{\text{CNTP}}=V\times \frac{T_{\text{CNTP}}}{T}\times \frac{P+∆H-P_{\mathrm{sol}}}{P_{\text{CNTP}}}$$

where V_STP_ is corrected biogas volume (Nm^3^); V is biogas volume measured in the gas holder (m^3^); T is ambient temperature (K); T_STP_ is standard temperature (273.15 K); P_STP_ is standard pressure (100 kPa); ΔH is gas holder height (m); P_sol_ is vapor pressure of the solution (calculated using the Goff–Gratch equation); ambient atmospheric pressure is 0.937 atm.

The amount of methane dissolved in the effluent was determined by the syringe headspace method, as described by Czepiel et al. (1993). The gas was sealed in glass bottles and sent to the laboratory for analysis in the FID‐equipped gas chromatograph (GC‐2014, Shimadzu Corporation). For calculating the dissolved methane concentration and COD load, the equation developed by de Souza (2010) was used, presented in Table 2.

**TABLE 2:** Equations used to calculate the portions of the COD mass balance.

Portions	Equations	Observations
COD_inf_	${\mathrm{COD}}_{\inf}=F\times C_{\inf-\mathrm{tot}}$	COD_inf_ is daily mass of COD at the inlet to the biodigester (kg day^−1^); F is influent flowrate (m^3^ day^−1^); C_inf‐tot_ is concentration of total COD in the influent (kg m^−3^).
${\mathrm{COD}}_{{\mathrm{CH}}_{4}}$ ^a^	$F_{{\mathrm{CH}}_{4}-\mathrm{STP}}=F_{\text{biogas}-\mathrm{STP}}\times \%{\mathrm{CH}}_{4}$ ${\mathrm{COD}}_{{\mathrm{CH}}_{4}}=\frac{F_{{\mathrm{CH}}_{4}-\mathrm{STP}}\times P\times K_{\mathrm{COD}}\times 1,000}{R\times \left(273+T\right)}$	F_CH4‐STP_ is volumetric production of methane (m^3^ day^−1^); F_biogas‐STP_ is volumetric production of biogas (m^3^ day^−1^); % CH_4_ is methane content in biogas; ${\mathrm{COD}}_{{\mathrm{CH}}_{4}}$ is daily COD mass converted into methane (kg COD day^−1^); T is operational temperature of the reactor (°C); atmospheric pressure (0.937 atm); K_COD_ is COD of one mole of CH_4_ (0.064 kg ${\mathrm{COD}}_{{\mathrm{CH}}_{4}}$ mol^−1^); R is gas constant (0.08206 atm L mol^−1^ K^−1^).
COD_sludge‐ret_ ^b^	${\mathrm{COD}}_{\text{sludge}-\mathrm{ret}}={\mathrm{COD}}_{\inf}\times \eta_{\mathrm{rem}}\times Y\times C\times L$	COD_sludge‐ret_ is daily COD converted into anaerobic sludge and retained in the reactor (kg day^−1^); COD_inf_ is daily mass of COD at the inlet to the biodigester (kg day^−1^); η_rem_ is COD removal efficiency in the biodigester (%); Y is conversion rate of removed COD into biomass (0.15 gVSS gCOD_removed_^−1^) C is COD/VSS ratio of the sludge(1.42) L is sludge retention rate in the biodigester (HF_w_ is 0.4 and HF_w/o_ is 0.8) (Neuner et al. 2024)
COD_sludge‐eff_	${\mathrm{COD}}_{\text{sludge}-\mathrm{eff}}=F\times \left(C_{\mathrm{eff}-\mathrm{tot}}-C_{\mathrm{eff}-\mathrm{sol}}\right)$	COD_sludge‐eff_ is daily COD mass converted into biomass and lost in the effluent (kg day^−1^); C_eff‐tot_ is concentration of total COD in the effluent (kg m^−3^); C_eff‐sol_ is concentration of soluble COD in the effluent (kg m^−3^);
${\mathrm{COD}}_{{\mathrm{CH}}_{4}-d}$ ^a^, ^b^	${\left[{\mathrm{CH}}_{4}\right]}_{d}=\frac{{\left[{\mathrm{CH}}_{4}\right]}_{g}\times \left[d\times V_{\mathrm{gas}}+\left(P-P_{V}\right)\times K_{H}\times V_{L}\right]\;}{V_{L}}$ ${\mathrm{COD}}_{{\mathrm{CH}}_{4}-d}={\left[{\mathrm{CH}}_{4}\right]}_{d}\times F\times f_{{\mathrm{CH}}_{4}}$	[CH_4_]_d_ is concentration of dissolved methane in the liquid effluent (kg m^−3^); [%CH_4_]_g_ is percentage concentration of methane in the headspace of the syringe (%); d is methane density (mg L^−1^, as a function of ambient temperature); V_gas_ is headspace volume in the syringe (30 mL); P_V_ is water vapor pressure (atm, as a function of ambient temperature); K_H_ is Henry's law constant for methane (mg L^−1^.atm^−1^, as a function of ambient temperature and pressure); V_L_ is volume of liquid in the syringe (30 mL); ${\mathrm{COD}}_{{\mathrm{CH}}_{4}-d}$ is daily COD mass converted to methane dissolved in the effluent (kg day^−1^); f_CH4_ is conversion factor of methane mass into; COD mass (4 kg COD/kg CH_4_).
COD_eff_	${\mathrm{COD}}_{\mathrm{eff}}=F\times C_{\mathrm{eff}-\mathrm{sol}}$	COD_eff_ is daily COD mass not converted and lost in the effluent (kg day^−1^) C_eff‐sol_ is concentration of soluble COD in the effluent (kg m^−3^)

^a^ Lobato et al. (2012).

^b^ de Souza (2010).

The theoretical solubility of methane was calculated based on Henry's law, considering the methane content in biogas (gas partial pressure) and the ambient pressure and temperature.

Finally, the hydrolysis and methanogenesis percentage in the biodigesters were calculated according to Equations (2) and (3) provided by Ribera‐Pi et al. (2020), to evaluate the efficiency of the impeller blades in anaerobic digestion.

(2) $$H\;\left(\%\right)=\left(\frac{{\mathrm{CH}}_{4}\;\mathrm{as}\;\mathrm{COD}+s{\mathrm{COD}}_{\text{effl}}-s{\mathrm{COD}}_{\inf}}{t{\mathrm{COD}}_{\inf}-s{\mathrm{COD}}_{\inf}}\right)\times 100$$

where *H* (%) is hydrolysis percentage; ${\mathrm{CH}}_{4}\mathrm{as}\;\mathrm{COD}$ is CH_4_ load as COD (g day^−1^); $s{\mathrm{COD}}_{\text{effl}}$ is effluent soluble COD (g day^−1^); $s{\mathrm{COD}}_{\inf}$ is influent soluble COD (g day^−1^); $t{\mathrm{COD}}_{\inf}$ is influent total COD (g day^−1^); $s{\mathrm{COD}}_{\inf}$ is influent soluble COD (g day^−1^).

(3) $$M\;\left(\%\right)=\left(\frac{{\mathrm{CH}}_{4}\;\mathrm{as}\;\mathrm{COD}}{t{\mathrm{COD}}_{\inf}}\right)\times 100$$

where *M* (%) is methanogenesis percentage; ${\mathrm{CH}}_{4}\mathrm{as}\;\mathrm{COD}$ is CH_4_ load as COD (g day^−1^); $t{\mathrm{COD}}_{\inf}$ is influent total COD (g day^−1^).

### 2.4 COD Mass Balance

**FIGURE 2:**
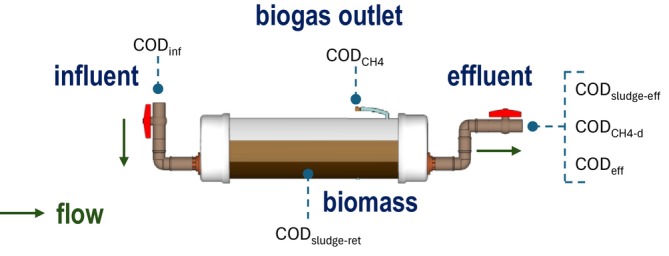
Portions of the COD mass balance in horizontal flow biodigesters treating swine wastewater.

The mass balance in the reactors was given by the quantification of organic matter, in terms of COD, considering the following portions: (i) influent COD (COD_inf_), (ii) COD converted into methane in biogas (${\mathrm{COD}}_{{\mathrm{CH}}_{4}}$), (iii) COD converted into anaerobic sludge and retained in the reactor (COD_sludge‐ret_), (iv) COD converted into sludge and lost with the effluent (COD_sludge‐eff_), (v) COD converted into methane and dissolved in the effluent (${\mathrm{COD}}_{{\mathrm{CH}}_{4}-d}$), and (vi) COD not converted and lost in the effluent (COD_eff_) (Lobato et al. 2012). In this study, the proportion of COD used by sulfate‐reducing bacteria was not considered. The COD/SO_4_^2−^ fraction was not considered in this study because sulfate reduction in SW is not favored, given the typically high COD/SO_4_^2−^ ratio characteristic of this type of effluent (Oliveira et al. 2021). Equation (4) presents the general equation of the balance, and the considered portions are illustrated by the scheme presented in Figure 2. The equations used to calculate each portion are shown in Table 2.

(4) $${\mathrm{COD}}_{\inf}={\mathrm{COD}}_{{\mathrm{CH}}_{4}}+{\mathrm{COD}}_{\text{sludge}-\mathrm{ret}}+{\mathrm{COD}}_{\text{sludge}-\mathrm{eff}}+{\mathrm{COD}}_{{\mathrm{CH}}_{4}-d}+{\mathrm{COD}}_{\mathrm{eff}}$$

where ${\mathrm{COD}}_{\inf}$ is daily mass of influent COD (kg day^−1^); ${\mathrm{COD}}_{{\mathrm{CH}}_{4}}$is daily mass of COD converted into methane in biogas (kg day^−1^); ${\mathrm{COD}}_{\text{sludge}-\mathrm{ret}}$ is daily mass of COD converted into sludge and retained in the reactor (kg day^−1^); ${\mathrm{COD}}_{\text{sludge}-\mathrm{eff}}$ is daily mass of COD converted into sludge and lost with the effluent (kg day^−1^); ${\mathrm{COD}}_{{\mathrm{CH}}_{4}-d}$ is daily mass of COD converted into methane dissolved in the effluent (kg day^−1^); ${\mathrm{COD}}_{\mathrm{eff}}$ is daily mass of effluent COD (kg day^−1^).

### 2.5 Statistical Analysis

The data were evaluated using descriptive statistical analysis, considering the mean, median, minimum and maximum values, standard deviation, and removal efficiency (%), calculated as the ratio between the difference between inlet and outlet concentrations and the inlet concentration, expressed as a percentage. Box‐and‐whisker plots (boxplots) were used to present hydrolysis and methanogenesis percentage. Sankey diagrams were generated using the web‐based tool SankeyMATIC (2026) to illustrate the mean distribution of mass balance fractions.

In addition, pH, temperature, total alkalinity, IA/PA ratio, COD (total and soluble), solids (total and volatile), and biogas and methane productions were analyzed by means of the Mann–Whitney statistical test, with Bonferroni correction (at the significance level of 5%), to verify the differences between the reactors in Phases I and II. Statistical analyses were performed using R, version 4.2.2.

## 3 Results

### 3.1 Stability and Performance of the HF Biodigesters

Table 3 shows the characteristics of the effluent at the outlet of the reactors in Phases I and II. The effluent pH of the reactors showed no statistical differences during Phase I, and the medians were above 7.10. However, in Phase II, the pH of the stirred reactor (HF_w_) was significantly distinct from the pH of the reactor without stirring (HF_w/o_), but both medians were ≥ 6.90. The TA in the reactors showed similar behavior and no significant difference during Phase I, with medians above 2000 mg L^−1^CaCO_3_. However, in Phase II, the median TA values of HF_w_ was significantly different from those of the HF_w/o_. The IA/PA ratio of the reactors was similar in both phases (median < 0.40), with no statistical differences throughout the operation.

**TABLE 3:** Characterization of the HF biodigesters effluent.

Phase	HF	Statistics	pH	TA	IA/PA	COD_t_	COD_s_	TS	TVS	TVS/TS	% Hydr.	% Met. Metanog
I	With mixing	*N*	50	17	17	17	18	17	17	17	6	6
		Mean	7.10	2284.94	0.31	16,945	462	17,505	11,658	66.61	13.4	21.5
		Median	7.11^a^	2295.84^a^	0.27^a^	14,228^b^	488^b^	17,970^b^	11,830^b^	65.96^b^	8.4^b^	20.0^b^
		Max	7.29	3185.48	0.66	30,900	611	23,730	16,460	82.85	31.9	35.3
		Min	6.87	1680.84	0.07	4450	239	9050	5810	62.15	6.8	15.4
		SD	0.08	386.06	0.14	7880	107	4264	2906	4.43	9.1	6.6
		Efficiency (%)				−41.5	73.1	−20.4	−5.8			
	Without mixing	*N*	50	17	17	17	18	17	17	17	6	6
		Mean	7.13	2111.60	0.29	4104	579	6211	3912	62.21	29.4	34.6
		Median	7.12^a^	2123.65^a^	0.26^a^	4794^a^	581^a^	6195^a^	3870^a^	60.69a	31.1^a^	35.0^a^
		Max	7.32	2525.42	0.59	7874	747	9650	6835	85.54	45.7	45.4
		Min	6.92	1740.87	0.12	925	396	2820	1360	48.23	13.4	24.3
		SD	0.09	156.77	0.13	1824	102	1807	1390	9.33	11.4	7.7
		Efficiency (%)				64.8	66.4	58.8	65.7			
II	With mixing	*N*	49	14	14	14	14	14	14	14	5	6
		Mean	6.88	1471.85	0.32	6399	519	8440	5671	66.78	13.3	17.9
		Median	6.90^b^	1446.66^b^	0.32^a^	6002^a^	533^a^	8500^a^	5720^a^	66.82^b^	11.8^a^	18.3^a^
		Max	7.03	1860.93	0.50	10,066	638	15,780	10,270	74.48	28.8	32.6
		Min	6.67	1231.20	0.19	2927	393	3630	2110	58.13	2.7	3.5
		SD	0.08	182.20	0.08	1935	84	3161	2151	3.31	9.0	10.6
		Efficiency (%)				52.9	65.2	42.8	50.8			
	Without mixing	*N*	49	14	14	14	14	14	14	14	7	7
		Mean	6.96	1613.05	0.26	5504	510	7222	4544	62.16	21.8	26.8
		Median	6.97^a^	1610.69^a^	0.26^a^	4743^a^	509^a^	6880^a^	4637^a^	63.34^a^	20.0^a^	23.3^a^
		Max	7.08	1785.24	0.44	10,159	621	11,110	7045	72.18	40.3	46.5
		Min	6.76	1415.88	0.14	2172	412	3925	1710	38.56	7.2	10.7
		SD	0.07	95.11	0.08	2417	61	2218	1548	7.15	9.9	10.9
		Efficiency (%)				56.3	65.1	53.8	63.8			

*Note:* Different letters represent significant differences between the treatments, by the Mann–Whitney test.

Abbreviations: COD_s_, soluble chemical oxygen demand (mg L^−1^); COD_t_, total chemical oxygen demand (mg L^−1^); IA/PA, intermediate alkalinity and partial alkalinity ratio; n, number of samples; SD, standard deviation; TA, total alkalinity (mg L^−1^ CaCO_3_); TS, total solids (mg L^−1^); TVS, total volatile solids (mg L^−1^). TVS/TS, ratio of volatile solids to total solids (%).

**FIGURE 3:**
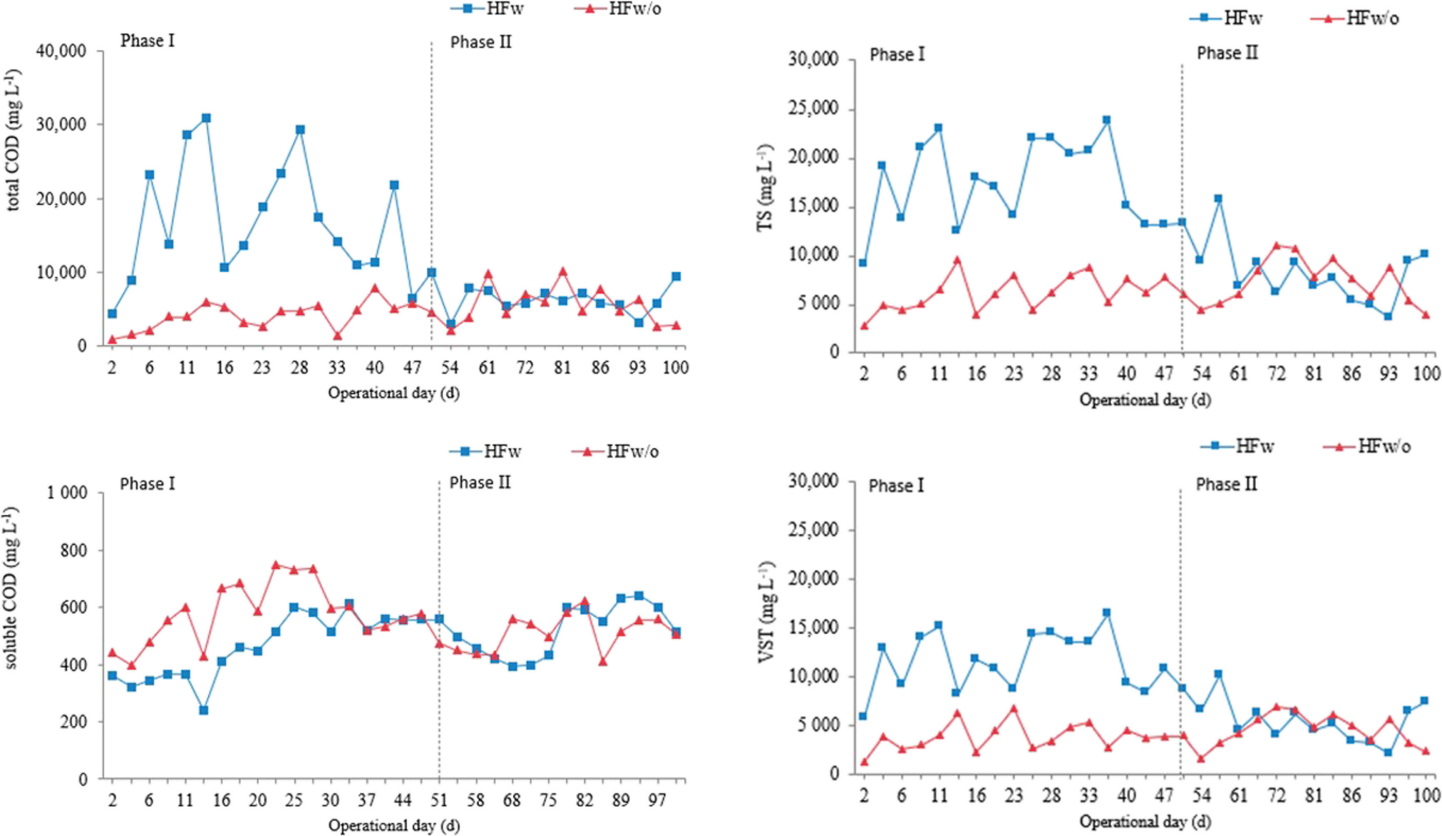
Concentrations of COD_t_ and COD_s_ and TS and TVS for the HF reactors (HF_w_: with stirring; HF_w/o_: no stirring).

Figure 3 shows the concentrations of total and soluble COD and total and volatile solids during the operation of the reactors.

First, it is worth noting that the SW used in the experiment consists of highly biodegradable substrate (TVS/TS = 78.8%) with a high particulate fraction (87.9%). Thus, during Phase I, the removal efficiency of particulate organic matter (COD_t_, TS, and TVS) can be associated with the presence/absence of agitation. In this phase, HF_w/o_ showed positive efficiencies for COD_t_, TS, and TVS (> 58%), whereas the HF_w_ showed negative efficiencies for these parameters, with higher concentrations at the outlet than at the inlet, and significantly different from HF_w/o_. However, during Phase II, the concentrations of COD_t_, TS, and TVS in the HF_w_ effluent dropped. As a consequence, the removal efficiencies became positive, with values above 50%. At this stage, the median concentrations of the parameters showed no statistical differences between the reactors (*p* > 0.05).

After treatment, the HF_w_ effluent in both phases remained with a higher biodegradability based on the TVS/TS ratio (> 66.0%), compared to HF_w/o_ (< 63.5%) (*p* ≤ 0.05), with part of the active biomass being washed from the HF_w_ reactor by the operation of the impeller blades.

Finally, with respect to COD_s_ in Phase I, the HF_w_ effluent had lower concentrations (*p* ≤ 0.05) than HF_w/o_. In Phase II, the COD_s_ concentrations at the outlet of both HF_w_ and HF_w/o_ showed no significant difference.

**FIGURE 4:**
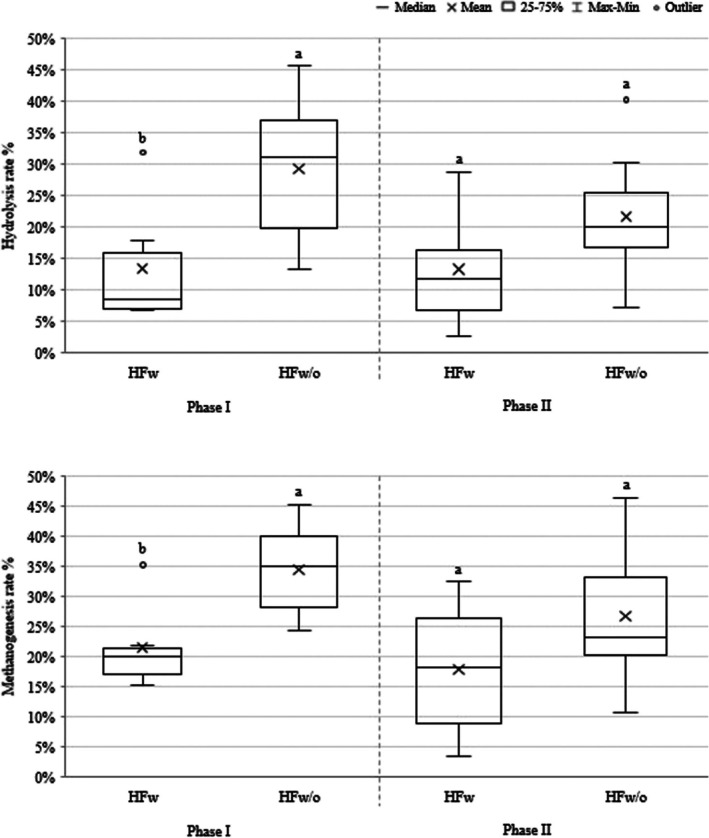
Hydrolysis and methanogenesis percentage in the HF biodigesters. Different letters represent significant differences between the treatments, by the Mann–Whitney test.

Hydrolysis and methanogenesis percentages were higher for HF_w/o_ in Phase I (*p* ≤ 0.05), and despite the continuation of high values in Phase II, there was no statistical difference between the reactors, as shown in Figure 4. In addition, methanogenesis was more prominent compared to hydrolysis, registering higher percentages throughout the operation.

### 3.2 Biogas and CH_4_ Yield

Table 4 presents biogas yields and CH_4_ content in biogas for each reactor, during the operation Phases I and II.

**TABLE 4:** Specific yield of biogas and methane (CH_4_) in anaerobic reactors during the operation phases.

Yield	Phase I		Phase II	
	HF_w_	HF_w/o_	HF_w_	HF_w/o_
L kg^−1^_TVSappl_	129.2 ± 48.9 (7)^b^	200.1 ± 56.4 (7)^a^	115.1 ± 46.8 (8)^b^	183.1 ± 55.3 (8)^a^
L kg^−1^_CODappl_	129.5 ± 26.8 (7)^b^	205.6 ± 37.1 (7)^a^	103.9 ± 58.5 (8)^a^	158.5 ± 59.1 (8)^a^
L_CH4_ kg^−1^_TVSappl_	88.8 ± 31.4 (6)^a^	139.7 ± 30.9 (6)^a^	73.2 ± 34.3 (6)^a^	120.0 ± 47.5 (7)^a^
L_CH4_ kg ^−1^_CODappl_	86.5 ± 26.9 (6)^b^	140.4 ± 31.2 (6)^a^	77.1 ± 40.0 (6)^a^	108.3 ± 44.8 (7)^a^
% v/v	65.2 ± 5.6 (6)^a^	65.5 ± 4.0 (6)^a^	56.9 ± 15.8 (6)^a^	64.2 ± 6.3 (7)^a^

*Note:* Mean ± SD (number of samples); different letters represent significant differences between the treatments in the same column, by the Mann–Whitney test.

Abbreviations: COD_appl_, COD applied to the reactor; HF_w_, HF with stirring; HF_w/o_, HF without stirring; TVS_appl_, TVS applied to the reactor.

Biogas and CH_4_ yield in terms of introduced organic matter (applied TVS and COD) were higher for HF_w/o_, compared to HF_w_. The biogas composition, in terms of methane (% v/v), showed medians above 60% with no significant differences for both reactors and operation phases (I and II). In the anaerobic digestion of SW, the CH_4_ content in the biogas is expected to reach values between 60% and 70% (Nagy and Wopera 2012).

### 3.3 COD Mass Balance

**FIGURE 5:**
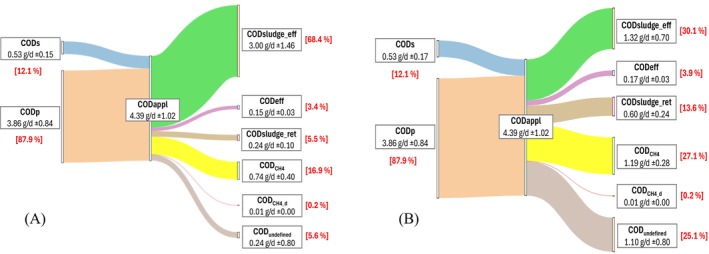
Sankey diagram of the COD balance in HF biodigesters (A) with stirring; (B) without stirring.

Figure 5 presents the variation of the mass balance portions in Sankey diagrams (%) for the reactors in Phase II, which was the more stable period of the operation.

As previously stated, the SW had a high particulate COD content, accounting for 87.9% of the substrate, whereas 12.1% of the COD was in its soluble form. At the outlet of the reactors, COD_s_ represented only 3.4% for HF_w_ and 3.9% for HF_w/o_.

In the HF_w/o_ biodigester, 27.1% of the organic matter applied to the system was converted into methane, while 13.6% of the total influent COD was retained as biomass. Conversely, in the HF_w_ reactor, although only 5.5% of the influent COD was associated with biomass retention, a methane production equivalent to 16.9% of the applied COD was achieved. Moreover, approximately 68.4% of the sludge was discharged with the effluent, representing the predominant fraction in the overall COD mass balance.

The COD corresponding to methane dissolved in the effluent represented the smallest fraction of the COD mass balance in both biodigesters, accounting for only 0.20% in each reactor. Theoretical methane solubility, estimated from biogas concentrations, was 11.7 mg L^−1^ for HF_w_ and 12.4 mg L^−1^ for HF_w/o_, whereas the experimentally measured concentrations were 8.9 and 7.5 mg L^−1^, respectively. Therefore, the methane concentrations observed in this study did not exceed the saturation limit (saturation degree < 1).

During the study period, the retained solids loading rate within the reactors were 1.35 g day^−1^  ± 0.4 of TVS and 1.70 g day^−1^  ± 0.7 of TVS for the stirred and nonstirred systems, respectively. Considering these variations, the solids accumulation in both reactors can be regarded as comparable.

## 4 Discussion

### 4.1 Stability of the Anaerobic Digestion in HF Biodigesters (Phases I and II)

Regarding the stability indicators—pH, alkalinity, and the IA/PA ratio—favorable conditions for anaerobic digestion and the development of methanogenic archaea were maintained in both operational phases. The average pH values ranged from 6.8 to 7.4, total alkalinity varied between 1000 and 3000 mg L^−1^ CaCO_3_, and the IA/PA ratio remained below 0.4 for both biodigesters (Martín‐González et al. 2013; Lourinho et al. 2020). Thus, despite the continuous local resuspension of active biomass in the HF_w_ reactor, the process remained stable without abrupt fluctuations.

Intermittent mixing in the HF_w_ reactor resulted in an effluent with a lower pH and higher IA/PA ratios compared to the HF_w/o_ reactor. This trend may be associated with the fact that mixing (i) can momentarily enhance hydrolysis and acidogenesis rates, leading to localized accumulation of volatile fatty acids (VFAs) and a temporary decrease in pH and alkalinity, and (ii) increases the efficiency of liquid–gas mass transfer, which probably intensified CO_2_ stripping and consequently caused the more pronounced and sudden pH variations observed (Lebranchu et al. 2017).

Wang et al. (2019) also reported lower pH values in a biodigester with intermittent stirring treating bovine wastewater, when compared with a nonmixed reactor (*p* ≤ 0.05). Similarly, in a UASB reactor treating SW, Rodrigues et al. (2014) observed that even during the initial operational period and under conditions of low COD removal efficiency, the average IA/PA ratio remained below 0.40. Therefore, both reactors were able to maintain stable anaerobic digestion performance throughout the entire operational period.

Since the synthetic wastewater was prepared by diluting only the solid fraction of manure collected from pig stalls in distilled water, the absence of urine, washing water, and feed residues may have contributed to the low alkalinity of the substrate. Therefore, it can be inferred that the alkalinity in both reactors was primarily dependent on that supplied by the SW itself. This hypothesis is supported by the gradual decline in total alkalinity observed throughout Phase II, although the buffering capacity of the systems was not compromised.

### 4.2 Performance of HF Biodigesters With and Without Stirring in Phase I

Before starting the operation, both reactors were acclimatized to the same conditions, that is, without mechanical stirring. In this period, biomass was retained so that the biodigesters maintained a similar initial amount of anaerobic sludge.

With the course of the operation and the continuous monitoring of the parameters over time, it became clear that there were two distinct operational phases, entitled Phases I and II. Phase I was defined by the removal of biomass that was close to the blades of the HF_w_, resulting in high concentrations of COD_t_, TS, and TVS in the effluent, unlike HF_w/o,_ which kept the solids through settling.

Among the stages of anaerobic digestion, hydrolysis is usually the speed‐limiting phase of the process for swine effluents (Li et al. 2019), since protein, one of its main components, is hydrolyzed at lower rates than carbohydrates (Zhang et al. 2023).

In a first stage (Phase I, 1–2 HRTs), the hydrolysis percentage in the mechanically mixed reactor was lower than in the nonmixed reactor, as confirmed by statistical analysis (Figure 3). The initially lower hydrolysis percentage observed in the mechanically mixed reactors can be attributed to several interacting mechanisms reported in the literature.

Mechanical mixing can provoke sloughing and dismantling of biofilms and microbial aggregates that are responsible for concentrated hydrolytic activity, temporarily reducing specific hydrolytic activity (Liu and Smith 2021). Mixing also redistributes fine particulate matter and may cause a relative washout of key hydrolytic populations, while rapid changes in liquid–gas transfer (e.g., CO_2_ stripping) and transient VFA accumulation alter the micro‐pH and enzyme environment, further depressing hydrolytic percentage until the microbial community re‐establishes (Ma et al. 2019; Khalil et al. 2021). Finally, homogenization removes localized hotspots of intense hydrolysis, producing a lower apparent local rate even if the volumetric conversion remains similar in the long term (Menzel et al. 2023). According to these authors, under plug‐flow regime, the operational stabilization of the system becomes more evident after approximately three HRTs, which was also verified here during Phase II (3–4 HRTs).

In Phase I, the biogas and CH_4_ yields in terms of TVS_appl_ and COD_appl_ were lower for HF_w_. It is worth noting that reduced biogas production can occur as a consequence of washing nondegraded organic matter, as well as active microorganisms necessary for hydrolysis and methanogenesis, and in extreme cases as a result of disturbance of the microbial community (Rico et al. 2011; Sun et al. 2019). Since HF_w_ was getting used to the mixing condition (Phase I), the washing of biomass may have restrained higher biogas and methane yields and caused the lower percentages of hydrolysis and methanogenesis.

### 4.3 Performance of HF Biodigesters With and Without Stirring in Phase II

With the establishment of Phase II, HF_w_ began to register TS, TVS, and COD removals similar to HF_w/o_. This shows that the HF_w_ adjusted to the stirring system, with defined areas for the concentration of biomass (space in between the blades) and distribution of the substrate (around the blades).

In an experiment with a bench‐scale CLB reactor with effluent recirculation, Bonugli‐Santos et al. (2022) showed that after 75 days of operation, the reactor achieved total COD removal efficiency of 82.6%. In other studies, total COD removal for reactors with intermittent stirring was in the range of 37%–57% (Karim et al. 2005; Wang et al. 2019), values close to those found in this research during Phase II. For the removal of solids in stirred reactors, Karim et al. (2005) obtained efficiencies ranging from 37% to 40% and 50% to 63% for TS and TVS, respectively. This shows the importance of longer periods for the consolidation of the anaerobic digestion process under mixing conditions (Menzel et al. 2023). In addition, the low VOLRs and high HRTs could also have contributed to higher efficiencies at the end of Phase II.

The conversion of organic matter into biogas was comparable between the two reactors, as evidenced by the absence of a significant difference in methane production (in terms of applied COD and TVS) (Table 4), by the hydrolysis and methanogenesis percentage during Phase II (Figure 4), and by the COD mass balance and its variation between the mixed (0.74 g day^−1^ ± 0.40) and nonmixed (1.19 g day^−1^ ± 0.28) reactors. In terms of biogas composition, the results were similar for both systems, with CH_4_ concentrations exceeding 60%. Yang and Deng (2020) reported comparable findings when analyzing the methane content in reactors operated with and without mixing.

In addition, it was evident that mechanical mixing promoted a greater loss of sludge in the effluent (68.4% of the applied COD), compared to 30.1% in the nonmixed reactor. This behavior can be interpreted as beneficial, since biogas production was not compromised by mixing. Moreover, the overall COD removal efficiencies were 56% and 53% for the mixed and nonmixed systems, respectively. In compact or low‐buffer anaerobic bioreactors (CLBs), such a practice may be advantageous, as the excessive accumulation of anaerobic sludge can reduce the effective working volume, increase sludge handling costs, and alter hydrodynamic flow patterns (Alvarado et al. 2012). Therefore, maintaining a mixing intensity that prevents excessive sludge accumulation may reduce operational demands and extend the reactor's service life.

Considering the results obtained in the present study regarding the removal of excess sludge through mechanical mixing, it can be inferred that, in a full‐scale CLB with a flow rate of 51 m^3^ day^−1^, as reported by Sousa et al. (2022), and an influent COD of 5504 mg L^−1^ (present study), the deposition of approximately 38 m^3^ year^−1^ of sludge (107.3 kg day^−1^; ρ_sludge_ = 1020 kg m^−3^) within the biodigester could be avoided. This condition would favor the operational performance of the biodigester, contribute to maintaining the HRT, and reduce the frequency of sludge removal operations.

Other favorable aspects of mechanical mixing in CLBs may be associated with (i) improved liquid homogenization, (ii) enhanced substrate–biomass contact, (iii) prevention of dead zones or excessive sedimentation (Lindmark et al. 2014; Neuner et al. 2024), and (iv) greater operational flexibility in defining specific phases and/or zones for hydrolysis and methanogenesis. Furthermore, understanding the limitations of mixing under the studied conditions—possibly related to excessive shear stress and sludge fragmentation—allows for the establishment of operational boundaries and alternative strategies, particularly regarding the application of mixing in anaerobic reactors treating low‐strength wastewater with a predominance of particulate organic matter applied to the reactor, as identified in this study.

The present study considered a two‐stage anaerobic reactor operated with mechanical mixing at 25 rpm. Further studies should be conducted to optimize biogas production under the proposed mixing strategy and to improve the efficiency of anaerobic digestion, thereby strengthening its sustainability as a green energy source, as also highlighted by Neuner et al. (2024).

Ma et al. (2019) evaluated the effect of mixing intensity on hydrolysis and acidification within the range of 30–120 rpm and concluded that mixing at 90 rpm was the most suitable condition. Operating digesters at low mixing intensities, although generally favorable for enhancing CH_4_ production and reducing energy demand, may impair the hydrolysis and acidification of suspended solids. Therefore, to better understand the influence of mixing on the anaerobic digestion process, more comprehensive studies are required, taking into account the simultaneous optimization of CH_4_ generation and the performance of hydrolysis and acidification stages.

The calculated specific power in the stirred reactor (≈0.035 W·m^−3^) is consistent with small‐scale bench reactors equipped with low‐speed agitators, as reported by Kaiser et al. (2016). This value is below the turbulent regimes typical of industrial bioreactors but is sufficient to ensure liquid homogenization.

Another finding of this study was that the methane produced in the reactors did not result in effectively high solubility, with concentrations remaining below the saturation levels predicted by Henry's law. The least representative fraction in the COD mass balance was the methane dissolved in the effluent (${\mathrm{COD}}_{{\mathrm{CH}}_{4}-d}$), accounting for only 0.2% of the total applied COD under both operational conditions. In this research, the use of an influent with high COD and solids concentrations, such as SW, combined with a reactor operated at a high HRT (HRT = 25 days), resulted in subsaturated dissolved methane concentrations and a negligible contribution of this fraction to the COD mass balance.

Yeo and Lee (2013) evaluated the effect of biomass retention, expressed as solids retention time (SRT), on dissolved methane concentration in a laboratory‐scale AnMBR, a high‐rate reactor operated at low HRT. Their results showed that higher SRTs can reduce the concentration of dissolved CH_4_ in the effluent while increasing the methane content in the biogas. Most studies on dissolved methane have been conducted using domestic sewage (Souza et al. 2011; Ramos et al. 2021), anaerobic reactors operated at low HRTs, and treating influents with low COD concentrations and organic loading rates. Under such conditions, the dissolved methane fraction tends to be highly representative in the COD mass balance (approximately 17%), and dissolved methane concentrations frequently reach supersaturation levels, typically 1.4 to 1.7 times the equilibrium concentration.

In this context, the hypothesis that wastewaters much more concentrated than domestic sewage would lead to even higher methane supersaturation (e.g., twofold or greater), and consequently to a similarly significant contribution of dissolved methane to the COD mass balance, was not confirmed by the results of the present study.

**FIGURE 6:**
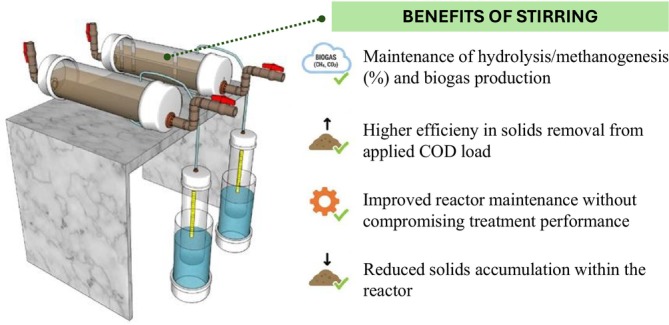
Main effects of the stirring mechanism in horizontal biodigesters.

Figure 6 presents a summary highlighting the main effects of the stirring mechanism in horizontal biodigesters on performance and organic matter conversion pathways, considering system stability conditions (Phase II).

Still, the intermittent mechanical stirring facilitated the removal of part of the stabilized sludge without compromising methane production. With this, the posttreatment units would be responsible for the retention of surplus sludge, to avoid the need of interventions in the reactor, such as the introduction of pumps for the removal of accumulated sludge at the base of the digesters.

## 5 Conclusion

This study evaluated the influence of mechanical stirring on the performance and stability of horizontal anaerobic biodigesters treating SW. Stability indicators confirmed stable anaerobic digestion in both operational conditions, demonstrating that intermittent mixing, despite slightly lower pH values and higher IA/PA ratios, did not compromise overall process stability. Mixing also did not negatively affect biochemical processes, maintaining hydrolysis and methanogenesis efficiencies as well as biogas yield and quality. The COD mass balance enabled an integrated understanding of organic matter conversion pathways and showed that stirred operation reduced solids accumulation within the reactor. From an operational perspective, mechanical stirring proved effective in preventing sludge buildup while preserving treatment efficiency and energy recovery. These findings provide relevant insights for the optimization and scale‐up of horizontal anaerobic reactors, supporting the adoption of mixing strategies to enhance long‐term operational performance in anaerobic digestion systems.

## Author Contributions

**Gabrielle Oliveira Rosa da Cruz:** conceptualization, methodology, formal analysis, investigation, data curation, writing – original draft, writing – review and editing, project administration. **André Pereira Rosa:** conceptualization, methodology, validation, formal analysis, investigation, data curation, writing – review and editing, supervision, project administration, funding acquisition. **Izabelle de Paula Sousa:** methodology, formal analysis, investigation, data curation, writing – review and editing, project administration. **Cláudio Leite de Souza:** methodology, validation, supervision, writing – review and editing, funding acquisition. **Alisson Carraro Borges:** methodology, data curation, writing – review and editing, supervision, project administration, funding acquisition.

## Conflicts of Interest

The authors declare no conflicts of interest.

## Data Availability

Data are available on request. E‐mail address of the person that can be contacted with requests for data (andrerosa@ufv.br).
